# Regulation of Schwann cell proliferation and migration via miR-195-5p-induced Crebl2 downregulation upon peripheral nerve damage

**DOI:** 10.3389/fncel.2023.1173086

**Published:** 2023-07-03

**Authors:** Shiying Li, Wenshuang Wu, Jing Zhang, Yu Chen, Yumeng Wu, Xinghui Wang

**Affiliations:** ^1^Key Laboratory of Neuroregeneration of Jiangsu and Ministry of Education, Co-Innovation Center of Neuroregeneration, NMPA Key Laboratory for Research and Evaluation of Tissue Engineering Technology Products, Nantong University, Nantong, Jiangsu, China; ^2^Cancer Research Center Nantong, Affiliated Tumor Hospital of Nantong University, Nantong, Jiangsu, China

**Keywords:** miR-195-5p, Crebl2, Schwann cell, proliferation, migration, peripheral nerve injury

## Abstract

**Background:**

Schwann cells acquire a repair phenotype upon peripheral nerve injury (PNI), generating an optimal microenvironment that drives nerve repair. Multiple microRNAs (miRNAs) show differential expression in the damaged peripheral nerve, with critical regulatory functions in Schwann cell features. This study examined the time-dependent expression of miR-195-5p following PNI and demonstrated a marked dysregulation of miR-195-5p in the damaged sciatic nerve.

**Methods:**

CCK-8 and EdU assays were used to evaluate the effect of miR-195-5 on Schwann cell viability and proliferation. Schwann cell migration was tested using Transwell and wound healing assays. The miR-195-5p agomir injection experiment was used to evaluate the function of miR-195-5p *in vivo*. The potential regulators and effects of miR-195-5p were identified through bioinformatics evaluation. The relationship between miR-195-5p and its target was tested using double fluorescence reporter gene analysis.

**Results:**

In Schwann cells, high levels of miR-195-5p decreased viability and proliferation, while suppressed levels had the opposite effects. However, elevated miR-195-5p promoted Schwann cell migration determined by the Transwell and wound healing assays. *In vivo* injection of miR-195-5p agomir into rat sciatic nerves promote axon elongation after peripheral nerve injury by affecting Schwann cell distribution and myelin preservation. Bioinformatic assessment further revealed potential regulators and effectors for miR-195-5p, which were utilized to build a miR-195-5p-centered competing endogenous RNA network. Furthermore, miR-195-5p directly targeted cAMP response element binding protein-like 2 (Crebl2) mRNA via its 3′-untranslated region (3′-UTR) and downregulated Crebl2. Mechanistically, miR-195-5p modulated Schwann cell functions by repressing Crebl2.

**Conclusion:**

The above findings suggested a vital role for miR-195-5p/Crebl2 in the regulation of Schwann cell phenotype after sciatic nerve damage, which may contribute to peripheral nerve regeneration.

## 1. Introduction

Nerve injury represents a global health issue that causes organ denervation, paralysis, anesthesia, and sensory, motor and autonomic dysfunctions (Allodi et al., 2012; Min et al., 2021). Fortunately, peripheral nerves have a stronger regenerative capacity than axons in the central nervous system (Gu et al., 2014; He and Jin, 2016). This capability mainly relies on the substantial plasticity of the main glia in peripheral nervous system-Schwann cells (SCs) (Taveggia and Feltri, 2022; Wang et al., 2022). Neurons are supported physically and trophically by SCs, which form the myelin sheaths surrounding the axons, making them essential for maintaining their normal function. In damaged peripheral nerves, Schwann cells first undergo dedifferentiation into an immature phenotype, proliferate in large quantities, and engulf axon and myelin sheath fragments, creating a regeneration path for subsequent neurite regeneration (Jessen and Mirsky, 2016, 2021). Then, Schwann cells begin to move along and begin to envelop the regenerated axons, and undergo the process of redifferentiation and myelination (Rao and Pearse, 2016; Bosch-Queralt et al., 2023). A permissive microenvironment is produced by SCs to induce neuronal repair (Stoll and Muller, 1999; Clements et al., 2017; Stratton et al., 2018; Motta et al., 2019). Considering the important functions of SCs, it might be beneficial to modulate their proliferative and migratory abilities following peripheral nerve injury (PNI) to support axonal outgrowth and functional recovery.

MicroRNAs (miRNAs or miRs) are ∼22-nucleotide long non-coding RNAs (lncRNAs) with diverse biological roles. miRNAs bind to the 3′-untranslated regions (3′-UTRs) of their target mRNAs, promote mRNA degradation and/or post-transcriptionally represess gene expression (Ambros, 2004; Krol et al., 2010; Diener et al., 2022). Abundant expression of miRNAs is found in the peripheral nervous system. Furthermore, a large number of miRNAs exhibit differential expression post-PNI (Yu et al., 2015; Gu et al., 2022). Current evidence suggests that differentially expressed miRNAs influence Schwann cell behaviors, such as myelinophagy, proliferation, migration and myelination, with potential effects on peripheral nerve repair and regeneration (Yi et al., 2017; Wang et al., 2019; Shen et al., 2022).

Recently, roles for miR-195-5p have been reported in many diseases such as preeclampsia (Zhou et al., 2022), inflammatory bowel disease (Scalavino et al., 2022b), cerebral ischemia-reperfusion (I/R) injury (Ren et al., 2021) and colorectal cancer (Lin et al., 2019). miR-195-5p is a critical regulator of many biological events such as cell proliferation and invasion (Wang et al., 2021), oxidative stress (Xia et al., 2022) and redistribution of tight junctions (Scalavino et al., 2022a). Additionally, miR-195-5p constitutes a potential diagnostic marker in various diseases (Li et al., 2020; Hu et al., 2021). According to our previous miRNA sequencing study, miR-195-5p is regulated following sciatic nerve transection in rat sciatic nerve stump specimens (Yu et al., 2011), indicating a major role in PNI and neuronal repair. However, miR-195-5p’s role in PNI is relatively unclear, especially in altering SC functions. This study firstly assessed miR-195-5p amounts in injured nerve samples from rats with experimental PNI. Further, biological roles of miR-195-5p on peripheral nerve regeneration were investigated. To determine miR-195-5p regulators after PNI, lncRNAs and target mRNAs of miR-195-5p were examined by bioinformatics. The direct interaction of miR-195-5p with the 3′-UTR of Crebl2, a gene that encodes cAMP response element (CRE)-binding protein-like-2, and miR-195-5p-induced Crebl2 downregulation were shown by luciferase assay and gene quantitation, respectively. To assess whether miR-195-5p suppresses Crebl2 and impacts Schwann cell phenotype, we conducted experiments to determine miR-195-5p’s biological roles in peripheral nerve regeneration and its regulatory effects on Crebl2 in Schwann cells, and assessed how Schwann cell characteristics fluctuate based on miR-195-5p.

## 2. Materials and methods

### 2.1. Animal study and tissue preparation

The sciatic nerve was crushed as described in a previous report (Qiao et al., 2022; Zhang et al., 2022). In brief, 12 male Sprague-Dawley (SD) rats (180–220 g) underwent anesthesia, and a 3-mm segment of the sciatic nerve in the left hindlimb was crushed using hemostatic forceps. For identifying gene expression patterns after crush injury, sciatic nerve samples were obtained at 0, 1, 3, and 7 days post-injury. The other SD rats were used for examining the *in vivo* effect of miR-195-5p. miR-195-5p agomir or agomir control was applied to rat sciatic nerves as described previously with modifications (Yi et al., 2017; Shen et al., 2022). Briefly, the *in vivo* effect of miR-195-5p on rat sciatic nerve was evaluated by distributing 5 nmol agomir or agomir control (RiboBio, Guangzhou, Guangdong, China) dissolved in 5 μL saline, mixing with Matrigel matrix basement membrane (Corning, NY, USA) at a volume ratio of 1: 1 and injecting 2 days prior to and immediately following crush injury in the epineurium of rat sciatic nerve. At 3 days after crush injury, rats sciatic nerve stumps were immunostained for MBP, S100β and NF200.

Specific-pathogen free (SPF) SD rats were provided by the Experimental Animal Center of Nantong University in China. The animal experiments were approved by the Administration Committee of Experimental Animals, Jiangsu, China (No. S20200323-190), following the Institutional Animal Care Guidelines of Nantong Province, Jiangsu, China. The animals were housed under SPF conditions at 23 ± 2°C and 55 ± 5% relative humidity with a 12-h/12-h photoperiod and food and water available all day.

### 2.2. Schwann cell culture and transfection

The primary SCs were obtained from 1-day old SD rats’ sciatic nerves using anti-Thy1.1 antibody and rabbit complement (Sigma, St. Louis, MO, USA) to remove fibroblasts as described previously (Li et al., 2015). The primary SCs were cultured in Dulbecco’s modified Eagle’s medium (DMEM; Gibco, Grand Island, NY, USA) containing 10% fetal bovine serum (FBS; Invitrogen, USA), 1% penicillin and streptomycin cocktail (Invitrogen, Carlsbad, CA, USA), 2 μM forskolin (Sigma) and 10 ng/mL heregulin (Sigma) at 37°C in a humidified incubator containing 5% CO2. Using Lipofectamine RNAiMAX (Invitrogen), purified SCs underwent a 48-h transfection with miR-195-5p mimic (miR-195-5p), mimic control (MC), miR-195-5p inhibitor (anti-miR-195-5p), inhibitor control (IC), Crebl2 siRNA (siRNA-Crebl2) and scramble control siRNA (siRNA-con) (RiboBio), respectively at 37°C, following the manufacturer’s directions. For functional assays, SCs underwent treatment with 20 nM miRNA mimic, 100 nM miRNA inhibitor or 20 nM siRNA. Double-stranded sequences for miR-195-5p mimic were 5′-UAGCAGCACAGAAAUAUUGGC-3′ and 3′-GCCAAUAUUUCUGUGCUGCUA-5′; the single- stranded sequence of miR-195-5p inhibitor was 5′-GCCAAUA UUUCUGUGCUGCUA-3′. The siRNA sequences against Crebl2 (siRNA-1 [siG2009110426146358], GAGAGGAACTGGA AATGTA; siRNA-2 [siG2009110426147450], AGAACAAGTCT CAGCAGAA; siRNA-3 [siG2009110426148542], TCCCAAAGCT GGAAAGACA) were from RiboBio, as well as non-specific negative controls.

### 2.3. Quantitative real-time RT-PCR (qRT-pCR)

To prepare qRT-PCR samples, total RNA was purified with RNA-Quick Purification Kit (Yishan Biotechnology, Shanghai, China) and submitted to reverse transcription with Bulge-Loop miRNA qRT-PCR Starter Kit (RiboBio) or HiScript III RT SuperMix (Vazyme, Nanjing, Jiangsu, China). qRT-PCR utilized ChamQ SYBR qPCR Master Mix (Vazyme) on a StepOne Real-Time PCR System (Applied Biosystems, Foster City, CA, USA). A miR-195-5p Bulge-loop™ miRNA qRT-PCR Primer Set was from RiboBio. Crebl2 primers (sense 5′-TGGATGACAGCAAGGTGGTC-3′ and antisense 5′-GGACACCAACTCCTCCAAGT −3′) and GAPDH primers (sense 5′-ACAGCAACAGGGTGGTGGAC-3′ and antisense 5′-TTTGAGGGTGCAGCGAACTT-3′) were from Sangon Biotech (Sangon Biotech, Shanghai, China). The comparative 2^–ΔΔCt^ method was utilized for the analysis of data, normalized to U6 or GAPDH expression.

### 2.4. Cell viability assay

CCK-8 (Beyotime, Shanghai, China) was utilized for assessing cell viability. SCs were seeded in 96-well plates (2 × 10^4^/well in 100 μL medium) and further incubated with 10 μL CCK-8 solution for a period of 2 h after transfection. An optical density (OD) measuring device (BioTek, VT, USA) was used to read absorbance at 450 nm.

### 2.5. 5-Ethynyl-2′-deoxyuridine (EdU) proliferation assay

SCs after transfection with miR-195-5p mimic, miR-195-5p inhibitor and Crebl2 siRNA, and respective controls were seeded onto a poly-L-lysine-coated 96-well plate at 2 × 10^5^/mL. A Cell-Light EdU DNA Cell Proliferation Kit (Ribobio) was utilized to quantify Schwann cell proliferation as directed by the manufacturer. In brief, SCs were administered 50 μM EdU for 24 h, followed by fixation with 4% paraformaldehyde for 15 min at 25°C, staining with Apollo 567 and Hoechst 33342, and visualization under an Olympus BX51 fluorescence microscope (Olympus, Tokyo, Japan). Image-Pro Plus (Media Cybernetics, Silver Springs, MD, USA) was employed for quantitation, determining proliferation rates as EdU-positive cells/total cells.

### 2.6. Transwell migration assay

After transfection, SCs in 100 μl DMEM were placed in the superior compartment of a 6.5-mm Transwell with 8 μm pores (Costar, Cambridge, MA, USA) at 3 × 10^5^ cells/ml. Meanwhile, 500 μL complete DMEM medium containing 10% FBS was placed in the inferior compartment. SCs were incubated at 37°C for 24 h and the upper surface was cleaned. Cells that had traversed the membrane underwent a 15-min staining with 0.1% crystal violet at 25°C, followed by visualization under a DMR inverted microscope (Leica Microsystems CMS GmbH, Bensheim, Germany). Image-Pro Plus (Media Cybernetics) is used for quantification based on number of cells converted to grayscale.

### 2.7. Scratch assay

According to our previous study, we conducted the scratch assay (Shen et al., 2022). Briefly, 2 × 10^5^ cells/mL were added to plates containing a septum in the middle, which underwent incubation at 37°C. Each septum was removed after the cells reached confluence. SCs underwent an additional 9-h incubation and were visualized by phase-contrast microscopy (Leica Microsystems). Migration rates for SCs were obtained by measuring the remaining septal area with Image-Pro Plus (Media Cybernetics).

### 2.8. Immunofluorescence labeling

Sciatic nerve tissues were fixed with 4% paraformaldehyde, washed with PBS, and blocked with Immunol Staining Blocking Buffer (Beyotime), incubated with primary MBP antibody (Cell Signaling Technology, 78896S, Boston, MA, USA), S100β antibody (Sigma, S2532) and NF200 antibody (Sigma, N2912), and then incubated with Goat Anti-Rabbit secondary antibody conjugated to Cy3 (Proteintech, SA00009-2, Chicago, IL, USA) and Alexa Fluor 488 Goat Anti-Mouse IgG (Proteintech, SA00013-1) prior to mounting on slides. Fluorescent signals from different lasers were used to visualize tissue slides. Optical and epifluorescence microscopes (Axio Imager M2, Carl Zeiss Microscopy GmbH, Jena, Germany) were used to capture the images.

### 2.9. Plasmids and dual-luciferase reporter assay

TargetScan and MiRanda were utilized for the prediction of miR-195-5p’s binding sites on Crebl2. The 3′-UTR of Crebl2 underwent PCR amplification with rat genomic DNA as a template. Subcloning of PCR products was carried out directly downstream of the hRluc gene in pmiR-RB-REPORT™ (Ribobio) using *Xho*I and *Not*I to produce the dual-Luc-UTR reporter plasmid. Overlap PCR was performed to generate the 3′-UTR mutant reporter plasmid. DNA Sequencing was carried out for the verification of wild-type and mutant 3′-UTRs.

For the luciferase assay, exponentially growing HEK 293T cells in 96-well plates (10^4^ per well in 100 μL medium) underwent a 48-h co-transfection with 50 ng pmiR-RB-UTR and 100 nM miRNA mimic with Lipofectamine 6000™ (Beyotime). Then, a Dual-Glo luciferase assay system (Promega, Madison, WI, USA) was employed for measuring hluc and hRluc luciferase activities in cell lysates.

### 2.10. Bioinformatic analysis

TargetScan^1^ was employed for the prediction of upstream regulatory lncRNAs of miR-195-5p. Based on miRWalk 3.0,^2^ miRanda^3^ and the miRNA Target Prediction Database (miRdb),^4^ the downstream target mRNAs of miR-195-5p were predicted. A miR-195-5p-centered competing endogenous RNA (ceRNA) network was built from downstream lncRNAs and upstream mRNAs. Heatmaps for lncRNAs and mRNAs were obtained with meV 4.9.0^5^ based on previous sequencing data for sciatic nerve stumps at 0, 1, 4, 7, and 14 days following nerve crush injury (Yi et al., 2015). Sequencing data were deposited in the National Center for Biotechnology Information database (accession no. PRJNA394957 [SRP113121]) (Zhao and Yi, 2019).

### 2.11. Statistical analysis

GraphPad Prism 6.0 (GraphPad Software, Inc., La Jolla, CA, USA) was utilized for data analysis. In the analysis, none of the data were excluded. D’Agostinl-Pearson omnibus normality and Brown-Forsythe tests were firstly carried out before parametric and non-parametric tests, respectively. Student’s t test was used to analyze functional differences between Schwann cells after transfection miR-195-5p mimic or inhibitor. The one-way analysis of variance (ANOVA) with *post-hoc* Tukey’s honestly significant difference (HSD) test was used to analyze the mRNA expression of miR-195-5p in nerve stumps at different time points after sciatic nerve injury. *P* < 0.05 indicated statistical significance.

## 3. Results

### 3.1. miR-195-5p expression is decreased after sciatic nerve injury

**FIGURE 1:**
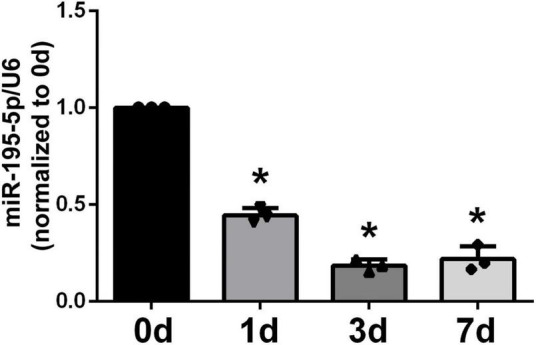
Temporal expression patterns of miR-195-5p in damaged nerve stumps following PNI. Following sciatic nerve crush injury in rats, miR-195-5p expression levels in damaged sciatic nerve specimens were determined by qRT-PCR at 0, 1, 3, and 7 days. *P < 0.05 versus control group (day 0) (*n* = 3, mean ± SEM; one-way analysis of variance with *post-hoc* Dunnett’s test was carried out to analyze the data). d: day(s).

We first assessed miR-195-5p expression in sciatic nerve specimens from rats following PNI by qRT-PCR. At 1 day post-PNI, miR-195-5p levels were about 2-fold lower than pre-injury levels (day 0, Figure 1). In comparison with the 0-day control level, miR-195-5p amounts were significantly reduced at the following time points, including 3 and 7 days.

### 3.2. miR-195-5p decreases SC proliferation

**FIGURE 2:**
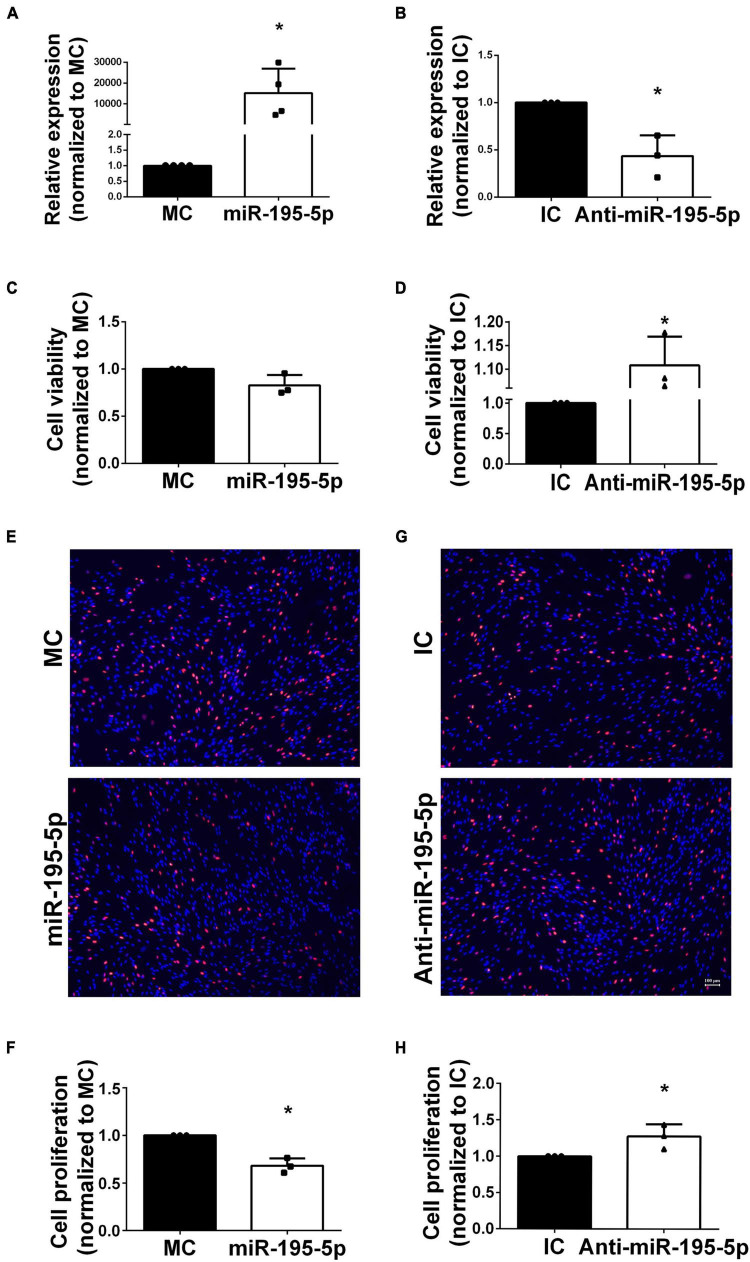
Effects of miR-195-5p on Schwann cell viability and proliferation. **(A)** Cells transfected with miR-195-5p mimic showed relatively enhanced miR-195-5p expression in comparison with MC transfection. *P < 0.05 versus MC group (mean ± SEM, *n* = 4; Student’s *t*-test). MC, mimic control. **(B)** Cells transfected with anti-miR-195-5p showed reduced miR-195-5p amounts in comparison with IC transfection. *P < 0.05 versus IC (mean ± SEM, *n* = 3; Student’s *t*-test). IC, inhibitor control. **(C)** Cell viability assessment in SCs after transfection with miR-195-5p mimic (miR-195-5p) or its control, and **(D)** with miR-195-5p inhibitor (anti-miR-195-5p) and its control by the CCK-8 assay. *P < 0.05 versus IC (mean ± SEM, *n* = 3; Student’s *t*-test). MC, mimic control; IC, inhibitor control. **(E,F)** Representative micrographs and proliferation rates of SCs transfected with miR-195-5p mimic (miR-195-5p) or its control. **(G,H)** Representative micrographs and proliferation rates of SCs transfected with miR-195-5p inhibitor (anti-miR-195-5p) or its control. Red, EdU-positive SCs; blue, Hoechst 33342 staining. Scale bar = 100 μm. *P < 0.05 versus MC or IC (mean ± SEM, *n* = 3; Student’s *t*-test). MC, mimic control; IC, inhibitor control.

Considering that SCs widely populate the sciatic nerve, they underwent transfection with miR-195-5p mimic or inhibitor before functional analyses *in vitro*. qRT-PCR was conducted on isolated SCs to determine transfection efficiency. In comparison with corresponding non-specific negative controls, SCs after transfection with miR-195-5p mimics (Figure 2A) and inhibitors (Figure 2B) showed markedly higher and lower miR-195-5p levels, respectively.

Transfected Schwann cells were examined for cell viability to assess miR-195-5p’s function in SCs and its associated effects on PNI. The CCK-8 assay showed miR-195-5p mimic-transfected cells had slightly decreased viability (Figure 2C), whereas miR-195-5p inhibitor-transfected SCs displayed increased viability (Figure 2D) compared with inhibitor controls. These findings suggested miR-195-5p slightly affected cell viability in SCs.

The EdU incorporation assay revealed miR-195-5p mimic transfection decreased Schwann cell proliferation (Figures 2E, F); conversely, miR-195-5p inhibitor transfection stimulated Schwann cell proliferation (Figures 2G, H).

### 3.3. miR-195-5p promotes migration in SCs

**FIGURE 3:**
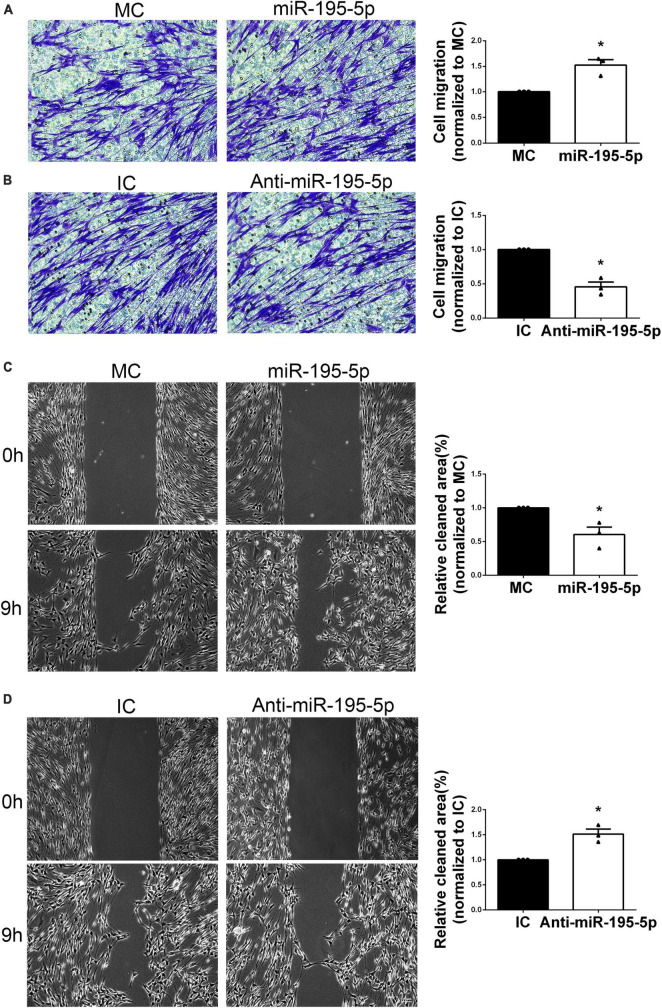
Effect of miR-195-5p on Schwann cell migration. Micrographs showing SCs that traversed the membrane of the Transwell membrane after transfection with **(A)** miR-195-5p mimic (miR-195-5p) or its control, and **(B)** miR-195-5p inhibitor (anti-miR-195-5p) or its control. Violet, migratory SCs. Quantification used histograms. *P < 0.05 versus MC or IC (mean ± SEM, *n* = 3; Student’s *t*-test). MC, mimic control; IC, inhibitor control. Scale bar = 50 μm. **(C)** Representative micrographs depicting SC wound healing and quantification of SC migration following transfection with miR-195-5p mimic (miR-195-5p) or its control (MC), and **(D)** miR-195-5p inhibitor (anti-miR-195-5p) or its control (IC). *P < 0.05 versus MC or IC (mean ± SEM, *n* = 3; Student’s *t*-test). MC, mimic control; IC, inhibitor control. Scale bar = 100 μm.

For the purpose of studying miR-195-5p’s impact on SC migration, SCs underwent transfection with miR-195-5p and Transwell migration assay. Significantly increased migratory potential was observed upon transfection with miR-195-5p mimic in comparison with the mimic control group, indicating miR-195-5p overexpression promoted SC migration (Figure 3A). Conversely, transfection with miR-195-5p inhibitor reduced the migratory potential of SCs versus the inhibitor control group (Figure 3B).

Additionally, the scratch assay was performed. After 9 h of culture following scratching, the miR-195-5p mimic group showed increased migratory capability versus control cells (Figure 3C). Conversely, SCs after transfection with miR-195-5p inhibitor showed impaired migratory capability (Figure 3D). The above findings suggested miR-195-5p promoted SC migration.

### 3.4. miR-195-5p modulates Schwann cell distribution, myelin preservation and axon elongation after peripheral nerve injury

**FIGURE 4:**
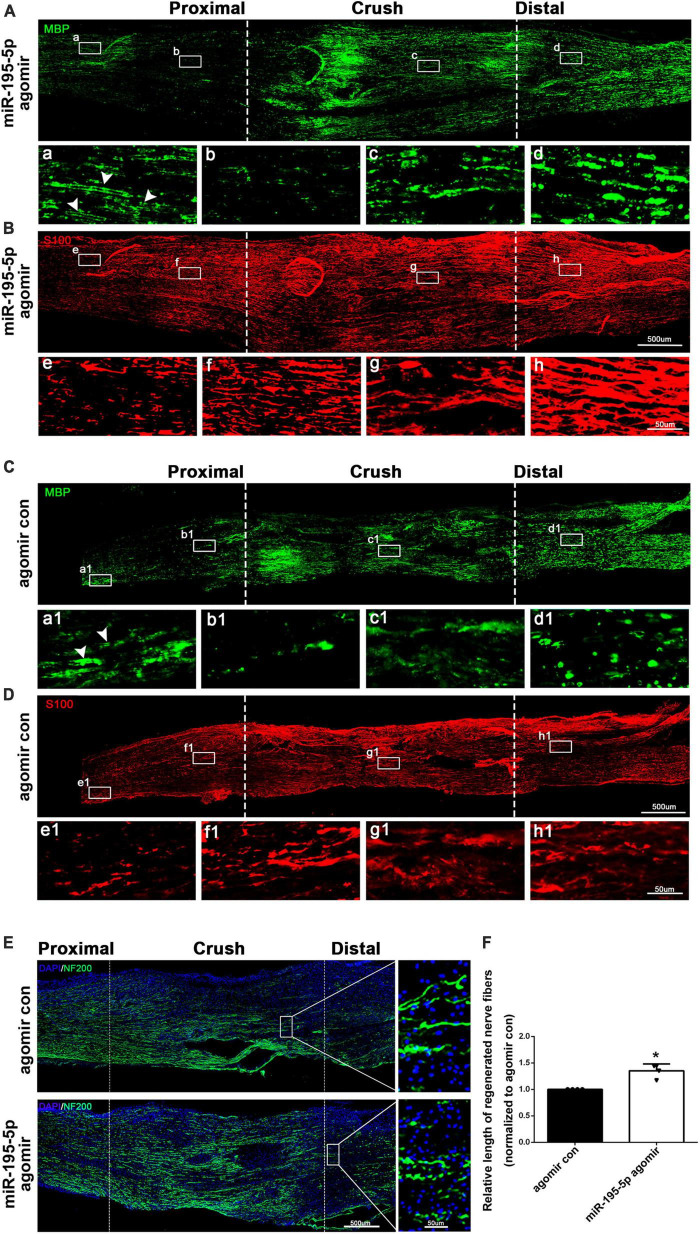
miR-195-5p affects Schwann cell distribution, myelin preservation and axon elongation *in vivo*. **(A,B)** Representative immunostaining of sciatic nerves of miR-195-5p agomir injected rats at 3 days after crush injury. Green color represents MBP staining and red color represents S100β staining. Crush sites are labeled with dashed lines. White boxed areas are displayed at a higher magnification below. White arrows indicate normal myelin tissue. Scale bars represent 500 μm in the main image and 50 μm in the magnified image. **(C,D)** Representative immunostaining of sciatic nerves of agomir control injected rats at 3 days after crush injury. Agomir con represents agomir control. Green color represents MBP staining and red color represents S100β staining. Crush sites are labeled with dashed lines. White boxed areas are displayed at a higher magnification below. White arrows indicate normal myelin tissue. Scale bars represent 500 μm in the main image and 50 μm in the magnified image. **(E)** Representative immunostaining of sciatic nerves of agomir control or miR-195-5p agomir injected rats at 3 days after crush injury. Green color represents NF200 staining and blue color represents nucleus staining. Crush sites are labeled with dashed lines. Boxed areas are demonstrated at a higher magnification on the right side. Scale bars represent 500 μm in the main image and 50 μm in the magnified image. **(F)** Quantification of the length of regenerated nerve fibers at 3 days after crush injury. *P < 0.05 versus agomir control (mean ± SEM, *n* = 4; Student’s *t*-test).

To investigate the *in vivo* effect of miR-195-5p, we directly applied miR-195-5p agomir to rat sciatic nerves after sciatic nerve injury. Immunostaining with anti-MBP and anti-S100β showed a notable correlation between Schwann cell distribution and myelin changes in the injured sciatic nerve. The analysis indicated that proximal nerve stumps situated away from the crush origin possessed a higher number of intact myelin sheaths (MBP) but a lower count of Schwann cells (S100β) [Figures 4A (a), 4B (e), 4C (a1), 4D (e1)]. However, in the proximity of the crush origin of the proximal nerve, there was a surge in the proliferation and dedifferentiation of Schwann cells, leading to their aggregation (S100β) in large quantities while myelin protein levels (MBP) witnessed a substantial decline [Figures 4A (b), 4B (f), 4C (b1), 4D (f1)]. Furthermore, as a result of axonal damage causing the collapse of myelin sheath, there was a significant accumulation of myelin debris in the injured segment and distal nerves [Figures 4A (c, d), 4C (c1, d1)].

The results of immunostaining using anti-MBP showed that the administration of miR-195-5p agomir via *in vivo* injection significantly preserved the myelin sheath across almost all segments of the injuried sciatic nerve, when compared to the control group. [Figures 4A (a–d), 4C (a1, b1, c1, d1)]. Immunostaining results using anti-S100β demonstrated that there was a higher distribution of Schwann cells in the proximal, crush, and distal nerve stumps in rats that received miR-195-5p agomir in comparison with rats injected with agomir control [Figures 4B (e–h), 4D (e1, f1, g1, h1)].

Sciatic nerve longitude sections were further immunostained with NF200, a marker of neuronal axons. Rats that received *in vivo* injections of miR-195-5p agomir showed significantly higher NF200 intensity and longer regenerated length of axons after 3 days of nerve injury compared to the agomir control (Figures 4E, F). These results suggest that miR-195-5p has an impact on Schwann cell distribution, as well as on myelin preservation after peripheral nerve injury, and furthermore stimulates the growth and elongation of axons.

### 3.5. miR-195-5p negatively regulates Crebl2 via direct interaction with its 3′-UTR

**FIGURE 5:**
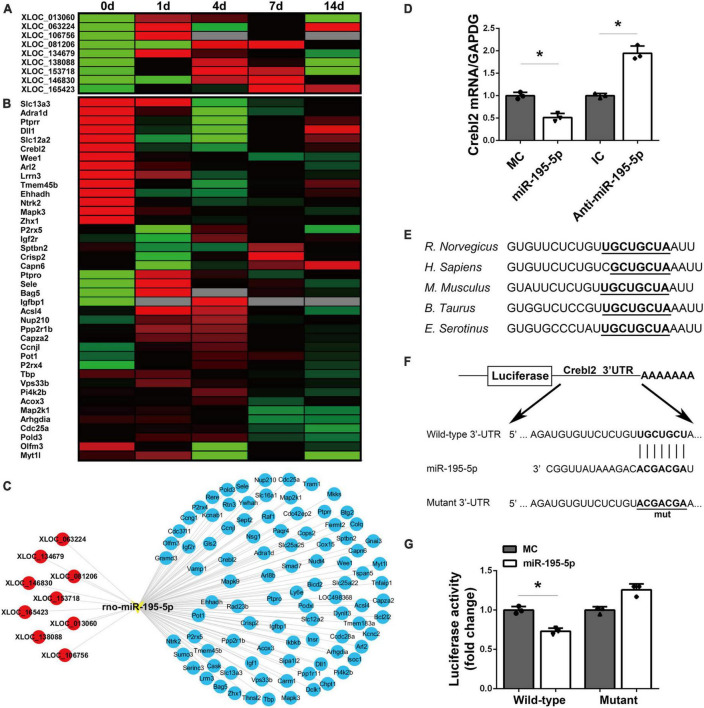
Identification of potential target mRNAs of miR-195-5p. **(A)** A heatmap showing the temporal expression patterns of lncRNAs in the miR-195-5p-centered ceRNA network for sciatic nerve stumps at 0, 1, 4, 7, and 14 days post-injury. Red, green, and gray indicate upregulation, downregulation, and no expression change, respectively. **(B)** A heatmap of the temporal expression patterns of mRNAs in the miR-195-5p-centered ceRNA network for sciatic nerve stumps at 0, 1, 4, 7, and 14 days post-nerve crush injury. ceRNA, competing endogenous RNA; lncRNA, long non-coding RNA; miRNA, microRNA. **(C)** Interactions among lncRNAs, miR-195-5p and target mRNAs in the miR-195-5p-centered ceRNA network. lncRNAs, the miRNA and mRNAs were labeled in red, yellow and blue, respectively. **(D)** Crebl2 mRNA amounts in SCs after transfection with miR-195-5p mimic (miR-195-5p) or its control (MC), and miR-195-5p inhibitor (anti-miR-195-5p) or its control (IC). *P < 0.05 versus MC or IC (mean ± SEM, *n* = 3; Student’s *t*-test). **(E)** Sequences of the predicted target site (1,250–1,257 bp) of miR-195-5p at the 3’-UTRs of Crebl2 in various species. **(F)** Constructions of wild-type and mutant pmiR-RB-UTR vectors. **(G)** Relative luciferase activities of 293T cells after transfection with WT or mutant pmiR-RB-UTR and miR-195-5p mimic (miR-195-5p) or mimic control (MC) *P < 0.05, mean ± SEM, *n* = 3; Student’s *t*-test.

The upstream lncRNAs and downstream mRNAs of miR-195-5p were examined. TargetScan prediction revealed 9 lncRNAs with potential interactions with miR-195-5p, including XLOC_013060, XLOC_063224, XLOC_106756, XLOC_081206, XLOC_134679, XLOC_138088, XLOC_153718, XLOC_146830 and XLOC_165423 (Figure 5A). miRWalk 3.0, miRanda, and miRdb predicted multiple potential miR-195-5p target mRNAs (Figure 5B). A miR-195-5p-centered lncRNA-miRNA-mRNA network was built (Figure 5C).

To verify the downstream target mRNAs of miR-195-5p, we screened the obtained target genes by qRT-PCR. qRT-PCR analysis demonstrated miR-195-5p mimic downregulated Crebl2 gene expression, which was upregulated by miR-195-5p inhibitor (Figure 5D). miRNA target prediction software confirmed miR-195-5p could interact with Crebl2 mRNA’s 3′-UTR. Furthermore, TargetScan analysis mapped miR-195-5p’s target site at 1,250–1,257 bp of the 3′-UTR of Crebl2 mRNA. The sequences of miR-195-5p’s binding site were conserved across species (Figure 5E). To determine whether Crebl2 is repressed by miR-195-5p via direct interaction with its 3′-UTR, the wild-type and mutant 3′-UTRs of Crebl2 were inserted downstream the luciferase reporter gene (Figure 5F). Co-transfection of the wild-type Crebl2-containing plasmid and miR-195-5p mimic resulted in decreased luciferase activity, while the mutant Crebl2-containing plasmid showed no effects (Figure 5G), suggesting a direct interaction between miR-195-5p and Crebl2 mRNA’s 3′-UTR.

### 3.6. miR-195-5p modulation of SC phenotypes is recapitulated by Crebl2 knockdown

**FIGURE 6:**
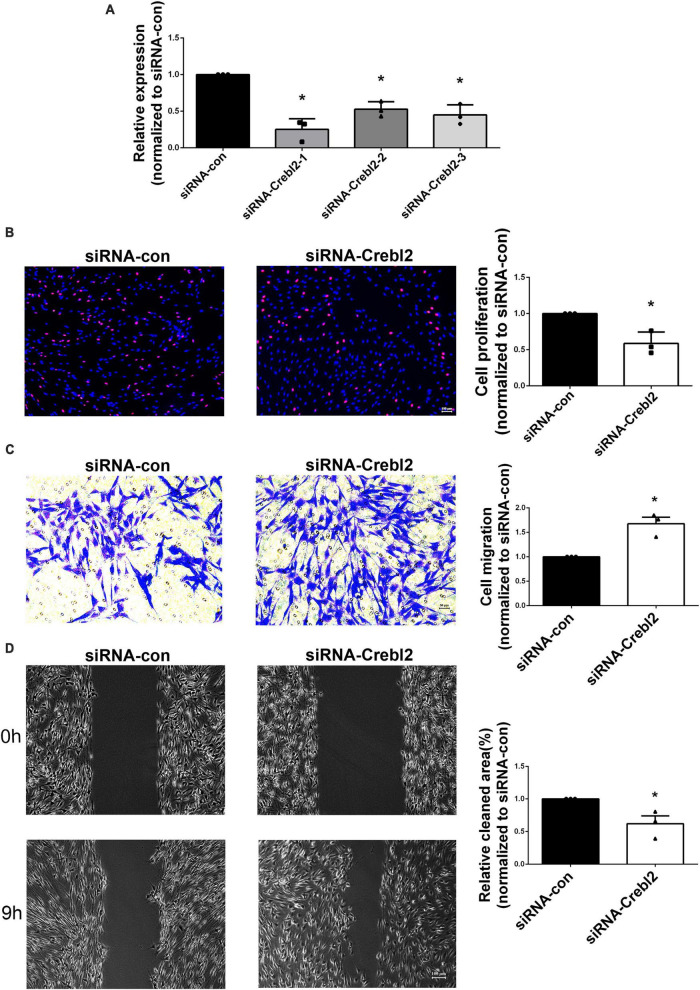
Crebl2 affects Schwann cell phenotype. **(A)** Interference efficiencies of three Crebl2 siRNAs. Effects of Crebl2 siRNA on Schwann cell **(B)** proliferation, **(C)** migration, and **(D)** wound healing. In panels **(B–D)**, scale bars are 100 μm, 50 μm and 100 μm, respectively. *P < 0.05 versus siRNA-con (mean ± SEM, *n* = 3; Student’s *t*-test). siRNA-con, control siRNA.

**FIGURE 7:**
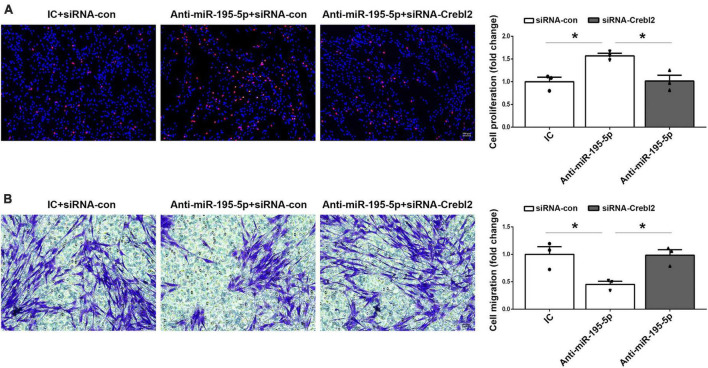
Crebl2 siRNA abrogates the effects of miR-195-5p inhibitor on Schwann cells. Rescue effect of Crebl2 siRNA in miR-195-5p inhibitor-regulated Schwann cell **(A)** proliferation and **(B)** migration. In panels **(A,B)**, scale bars are 100 μm and 50 μm, respectively. siRNA-con, control siRNA; IC, inhibitor control; Anti-miR-195-5p, miR-195-5p inhibitor. *P < 0.05, mean ± SEM, *n* = 3; Student’s *t*-test.

To investigate if miR-195-5p’s effects on SC proliferation and migration can be recapitulated by downregulating Crebl2, Crebl2 siRNA was transfected into primary SCs. As demonstrated by qRT-PCR, stable Crebl2 knockdown was achieved in SCs (Figure 6A). Similar to miR-195-5p overexpression, Crebl2 silencing resulted in suppressed cell proliferation (Figure 6B) and enhanced cell migration (Figures 6C, D). After transfection of primary SCs with Crebl2 siRNA and miR-195-5p inhibitor, miR-195-5p inhibitor-dependent elevation of cell proliferation and reduction of migration were markedly blunted by Crebl2 silencing (Figures 7A, B). Jointly, these data demonstrated Crebl2 acts as a functional modulator of miR-195-5p-mediated SC regulation.

## 4. Discussion

Mounting evidence suggests that dysregulated miRNAs have critical regulatory functions in multiple pathophysiological processes. Here, miR-195-5p, a miRNA with differential expression following nerve injury, regulated SC activation and modulated peripheral nerve regeneration.

miR-195-5p is involved in diverse neurological disorders such as acute ischemic brain damage (Zhu et al., 2021), subarachnoid hemorrhage (SAH)-induced cerebral vasospasm and brain injury (Tsai et al., 2021), Alzheimer’s disease (Liu et al., 2019) and intractable temporal lobe epilepsy (TLE) (Yu et al., 2021). Based on the above bioinformatic analysis of differentially expressed miRNAs in PNI, miR-195-5p was identified as a major upstream modulatory miRNA (Yu et al., 2011), suggesting miR-195-5p may affect SC behavior.

In this work, compared with pre-injury values, miR-195-5p levels in sciatic nerve stumps in the rat model of sciatic nerve injury were significantly decreased 1, 3, and 7 days following nerve injury. To determine the biological effects of miR-195-5p on Schwann cells by using cell proliferation assay, cell migration assay and sciatic nerve crush models. Functional results showed that miR-195-5p would decrease the proliferation of Schwann cells and increase the migration of Schwann cells and promote the axon regeneration. Subsequent mechanistic studies showed that Crebl2 knockdown recapitulated the effects of miR-195-5p inhibitor on SC proliferation and migration. These findings suggested miR-195-5p regulates SC activities in peripheral nerve regeneration by repressing Crebl2. Future studies may apply miR-195-5p to injured peripheral nerves, as a potential drug for PNI.

It should be noted that miR-195-5p expression was markedly decreased following PNI. Previously published reports (Zochodne, 2000) have suggested diverse stages following PNI, (e.g., early and late stages occurring 3 or 4 days apart post-PNI). SCs, which account for the majority of cells in the peripheral nervous system, have crucial biological functions in peripheral nerve repair (Nocera and Jacob, 2020). In peripheral nerve regeneration, however, SCs display two distinct phenotypes. In the early stage following PNI, SCs undergo dedifferentiation and proliferate, whereas they migrate and undergo redifferentiation at a later time point (Ness et al., 2013). Following PNI, proliferating SCs progressively move toward injured nerves, remove axons and myelin, and trigger regenerative events leading to axonal elongation (Nocera and Jacob, 2020). Therefore, miR-195-5p exhibits an expression trend whereby level alteration over time may impact both dedifferentiation and redifferentiation in SCs. By transfecting primary SCs with miR-195-5p mimic or inhibitor, we could comprehensively investigate miR-195-5p’s effects on SCs, and the mimic and inhibitor had opposite effects on SC proliferation and migration. As demonstrated above, miR-195-5p amounts start to decline after PNI, likely to enhance SC proliferation and dedifferentiation in the early stage of nerve injury to promote the regeneration of peripheral nerve injury. Then the miR-195-5p that to slightly up-regulated at later stage might benefit Schwann cell migration and redifferentiation. Multiple regulators are involved in cell proliferation and migration. However, many of them may exert distinct effects on cell proliferation and migration. We previously found that Mapt, a protein first downregulated following nerve injury and later upregulated, suppresses SC proliferation while enhancing migration (Yi et al., 2019). Consequently, miR-195-5p is likely to play different roles in SC proliferation and migration.

Furthermore, *in vivo* studies also show that altered expression of miR-195-5p modulates the Schwann cell phenotype and regulates axon regeneration. Immunostaining studies further demonstrated that miR-195-5p regulated Schwann cell distribution and myelin preservation in the damaged nerves. In the proximal nerve stumps, Schwann cells proliferate and dedifferentiate in lots, especially near injury sites, and then migrate to the injury sites and begin to redifferentiate with time (Ness et al., 2013). Our results show that a large number of Schwann cells were gathered and distributed in the proximal nerve near the injury site, while myelin protein levels were significantly reduced. However, at the injury site, the level of myelin protein increases. Our hypothesis is that the decrease in myelin protein expression may be attributed to the dedifferentiation of Schwann cells in the proximal nerve, while Schwann cells that migrate begin to redifferentiate, leading to an increase in myelin protein expression at the site of injury. At the same time, we observed a substantial accumulation of myelin debris in the injured segment and distal nerve stumps even after 3 days post-injury.

Next, lncRNAs and mRNAs with potential associations with miR-195-5p were assessed. Interestingly, miR-195-5p were well-conserved among diverse species, e.g., humans (*H. Sapiens*) and rats (*R. norvegicus*). Considering previously reported ceRNA networks for rats following PNI (Qian et al., 2018), this work assessed lncRNA-miR-195-5p-mRNA interactions, built a miR-195-5p-centered ceRNA network, and examined the time-dependent expression patterns of miR-195-5p-related lncRNAs and mRNAs in injured sciatic nerves from rats. The obtained heatmaps demonstrated inverse associations of miR-195-5p’s temporal expression profiles with all identified upstream lncRNAs, and miR-195-5p was also inversely correlated with its potential target gene Crebl2. Crebl2 encodes the cAMP response element binding protein-like 2 protein, which mostly regulates cellular metabolism and plays critical roles in adipogenesis and lipogenesis (Ma et al., 2011; Tiebe et al., 2019).

The involvement of Crebl2 in the nervous system remains unclear, although Crebl2 is considered the key individual factor inducing neural system development defects in offspring caused by copper exposure in parents (Tai et al., 2022). As shown above, sequencing data indicated Crebl2 expression first decreased and then increased in sciatic nerve stumps after nerve injury, which may be induced by downregulation of miR-195-5p. Besides luciferase and functional rescue assays, Crebl2 binding relationships with miR-195-5p were further validated. A deeper understanding of peripheral nerve injury could be gained by evaluating Crebl2 expression changes in Schwann cells.

Taken together, miR-195-5p regulates SC proliferation and migration. In addition, Crebl2 was identified as a miR-195-5p’s downstream functional target gene, providing insights into cellular metabolism and expanding understanding of the molecular changes associated with peripheral nerve injury.

## 5. Conclusion

In summary, miR-195-5p expression was dysregulated after PNI. In addition, miR-195-5p has the potential to affect the proliferative and migratory abilities of SCs via Crebl2-related downstream pathways. The current data might provide insights into miR-195-5p’s biological roles in the peripheral nervous system.

## Data availability statement

The datasets presented in this study can be found in online repositories. The names of the repository/repositories and accession number(s) can be found below: https://www.ncbi.nlm.nih.gov/, SRP113121.

## Ethics statement

The animal study was reviewed and approved by the Administration Committee of Experimental Animals, Jiangsu, China.

## Author contributions

XW: conceptualization. SL, WW, and JZ: methodology. JZ, YC, and YW: formal analysis. XW, SL, and WW: writing-original draft. XW and WW: writing-review and editing. XW and SL: supervision. All authors contributed to the article and approved the submitted version.

## Footnotes

1
http://www.targetscan.org/vert_71/

2
http://mirwalk.umm.uni-heidelberg.de/

3
http://mirtoolsgallery.tech/mirtoolsgallery/node/1055

4
http://mirdb.org/

5
https://sourceforge.net/projects/mev-tm4/files/mev-tm4/MeV%204.9.0/
